# Underestimated Centiloid Values in Amyloid PET: A Technical Report on Clinically Relevant Quantification Errors

**DOI:** 10.7759/cureus.97398

**Published:** 2025-11-21

**Authors:** Heii Arai

**Affiliations:** 1 Department of Psychiatry, Juntendo University, Tokyo, JPN; 2 Department of Psychiatry, ALZClinic Tokyo, Tokyo, JPN

**Keywords:** alzheimer’s disease, amyloid pet, anti-amyloid therapy, centiloid, florbetapir

## Abstract

Quantitative amyloid positron emission tomography (PET) interpretation using the Centiloid scale is increasingly adopted worldwide to guide eligibility and continuation of anti-amyloid monoclonal antibody therapy. However, discrepancies between visual and quantitative assessments occasionally occur, potentially leading to critical misjudgments in clinical decision-making. We present a representative 18F-florbetapir case in which cortical amyloid deposition was visually evident, yet the calculated Centiloid value was 0, falsely indicating a negative scan. This underestimation likely results from reference region selection: using the whole cerebellum (including white matter) lowers standardized uptake value ratios by approximately 7% compared with cerebellar gray matter, thereby decreasing Centiloid values. Consequently, patients with substantial amyloid burden may be incorrectly deemed ineligible for initiation or continuation of anti-amyloid therapy. Clinicians should therefore interpret Centiloid-based quantification with caution, always corroborating it with expert visual reads. Harmonization of reference region definitions and standardized reporting are urgently needed to prevent inappropriate treatment decisions and ensure the safe, effective use of disease-modifying therapies in Alzheimer's disease.

## Introduction

Since the regulatory approval of anti-amyloid monoclonal antibody therapies for Alzheimer's disease (AD), the clinical significance of amyloid positron emission tomography (PET) imaging has been further emphasized in both diagnostic and therapeutic settings. However, quantitative interpretation using the Centiloid (CL) scale may sometimes diverge from visual assessment, leading to potential misclassification in clinical decision-making.

At our institution, amyloid PET using 18F-florbetapir is routinely performed, and visual interpretation is prioritized as the primary determinant of amyloid positivity [1]. According to published visual-read criteria for florbetapir PET, a scan is considered visually positive if it shows (a) two or more cortical regions (each larger than a single gyrus) in which the gray-white matter boundary is indistinct or lost, or (b) any region where gray-matter uptake clearly exceeds adjacent white matter [1]. PET images are independently assessed by two licensed physicians, and discrepancies are resolved by consensus.

On the other hand, the CL scale provides a standardized quantitative framework for expressing amyloid PET burden on a 0-100 scale, enabling harmonized comparisons across tracers and institutions [2]. This system has been rapidly adopted in clinical and research settings worldwide, particularly in evaluating eligibility for anti-amyloid monoclonal antibody therapies such as lecanemab and donanemab.

Quantitative amyloid PET is first expressed as a standardized uptake value ratio (SUVR) computed as the mean uptake in a cortical composite volume-of-interest (VOI) divided by the mean uptake in a reference region (commonly, the whole cerebellum) within a fixed post-injection time window (e.g., 50-70 minutes for 18F-florbetapir). To place different tracers on a common scale, tracer-specific SUVRs are then linearly transformed to a Pittsburgh compound B (PiB)-equivalent SUVR using published calibration equations and finally converted to CL by anchoring 0 CL to the mean of young controls (YC) and 100 CL to the mean of typical AD patients:

$$CL = 100 \times \frac{(SUVR_{PiB\text{-}equivalent} - \mu_{YC})}{(\mu_{AD} - \mu_{YC})}$$

In this expression, μ (mu) represents the mean cortical SUVR for a given reference population. Specifically, μ_YC denotes the mean SUVR of YC who are assumed to have no amyloid deposition (defined as 0 CL), while μ_AD represents the mean SUVR of typical Alzheimer’s disease patients (defined as 100 CL). PiB serves as the reference tracer that defines this CL scale, providing a common calibration anchor so that SUVRs from other tracers (e.g., 18F-florbetapir, 18F-florbetaben, 18F-flutemetamol) can be linearly transformed into PiB-equivalent CL units. Because CL is a linear transform, values can be negative (below the YC mean) or exceed 100 (above the AD mean) [3].

However, in daily practice, discrepancies between visual reads and CL-derived quantitative assessments are occasionally observed. Previous studies have reported visual-quantitative discordance rates ranging from 5 to 15%, depending on the tracer and analysis pipeline [2]. Since current clinical guidelines consider a CL value below 24.1 to correspond to sparse or absent neuritic plaques, equivalent to a visually negative amyloid PET finding [4], quantitative underestimation may result in inappropriate clinical judgments.

Here, we report a representative 18F-florbetapir case in which the CL value was markedly underestimated despite visually clear cortical amyloid deposition. We further discuss potential technical causes and their implications for therapeutic decision-making.

## Technical report

All cases at our institution were evaluated with an amyloid PET imaging system (Biograph Horizon, Siemens Medical Solutions USA, Inc., Malvern, PA) using 18F-florbetapir. Reconstructions were performed using time-of-flight (TOF) measurements; pixel sizes in the axial plane and the slice thickness were 1.03 and 2.0 mm, respectively. Iterative reconstruction employed a three-dimensional ordered subset expectation maximization (OSEM) algorithm with eight iterations and 10 subsets. Attenuation correction was used, and post-filtering was applied using the Gaussian filter at 4 mm.

Quantitative analysis was conducted using a CL pipeline normalized to the whole cerebellum as the reference region [5]. For transparency, CL and Z-score computations followed the Global Alzheimer's Association Interactive Network (GAAIN) standard pipeline, implemented in a validated software package (AMYclz®, PDRadiopharma, Tokyo, Japan) based on SPM12 processing. In our institution, anatomical preprocessing and VOI definition were performed using PMOD v4.2 (PMOD Technologies, Zurich) with SPM12-based MR segmentation. The cortical composite and reference regions adhered to GAAIN-defined CL VOIs, and the software output included global CL values and regional Z-score maps aligned with MRI- or low-dose-CT-guided workflows.

**Figure 1:**
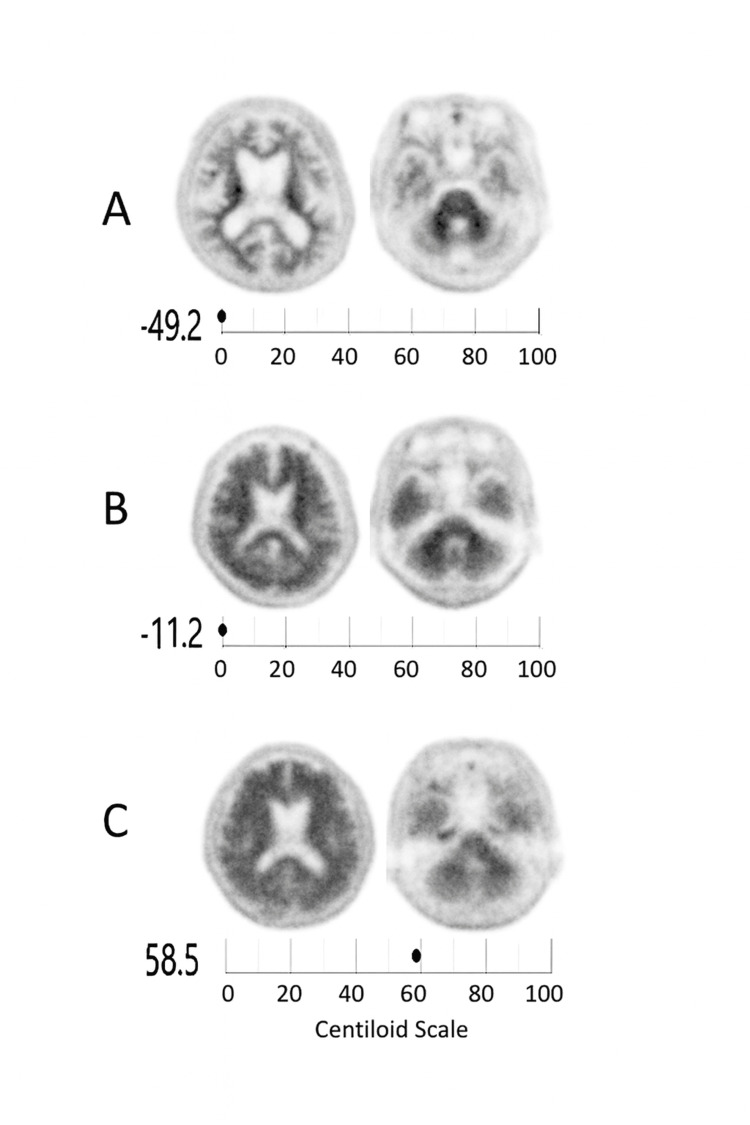
Representative amyloid PET images showing visual–quantitative discrepancies in Centiloid values Representative 18F-florbetapir PET scans from three patients evaluated with a Biograph Horizon PET/CT system (Siemens Healthineers) using time-of-flight acquisition and three-dimensional ordered-subsets expectation maximization (OSEM) reconstruction (eight iterations, 10 subsets; Gaussian filter 4 mm FWHM). Quantitative values were computed with the CL pipeline normalized to the whole cerebellum as the reference region using SPM12-based processing (AMYclz®, PDRadiopharma, Tokyo, Japan). (A) Visually and quantitatively negative (CL = –49.2, reported as 0 CL by the automated software); (B) visually positive but quantitatively negative (CL = –11.2, reported as 0 CL); and (C) both visually and quantitatively positive (CL = 58.5). Case B represents a 72-year-old male patient with a Mini-Mental State Examination (MMSE) score of 24 and a Clinical Dementia Rating–Global Scale (CDR-GS) score of 1. Each panel displays horizontal (axial) slices: the left column shows the cerebral hemispheric level around the lateral ventricles, while the right column shows the cerebellar level used for reference region assessment. The discrepancy in (B) reflects an underestimation when the whole cerebellum (including white matter) is used as the reference region, which lowers the SUVR and, in turn, the calculated CL value. Note: Although CL values can be negative because 0 CL is anchored to the mean of young controls [2], most clinical summary displays use a 0–100 range with integer reporting; consequently, negative values may appear as "0" in reports [6,7]. We therefore provide the raw computed value (–11.2 CL) together with the display value for transparency.

Three representative cases were selected (Figure 1). All three cases were evaluated with the same amyloid PET imaging system at our institution: (A) visually and quantitatively negative, (B) visually positive but CL = 0 (false-negative), and (C) visually and quantitatively positive.

In Case B, cortical amyloid uptake was visually prominent, yet the calculated CL value was 0, suggesting a false-negative result. As shown in the figure, the overall cerebellar uptake appeared higher than in Cases A and C, indicating greater non-specific retention in the reference region. Because cerebellar white matter demonstrates relatively higher non-specific tracer uptake than gray matter [8], inclusion of white-matter voxels within the reference region elevates its mean standardized uptake value and thereby lowers the cortical-to-cerebellar SUVR. Consequently, the resulting CL value becomes underestimated. The magnitude of CL reduction depends on the degree of non-specific uptake in the reference region; therefore, cases with higher cerebellar uptake, such as Case B, are more likely to yield underestimated CL values and thus false-negative classifications. In addition, the absence of a dedicated occipital-lobe ROI in the cortical composite in the validated software package may contribute to the underestimation of global CL values. However, when cerebellar gray-matter activity is relatively high, the whole-cerebellum reference can, conversely, lead to smaller bias or even lower SUVR and CL variability across individuals, underscoring that the impact of reference selection may vary depending on tracer kinetics and regional uptake characteristics.

These findings demonstrate that reference region definition can substantially influence SUVRs and CL values, particularly near the diagnostic threshold. This issue also reflects a broader challenge in amyloid PET quantification, ensuring consistency across analysis pipelines, reference region definitions, and software platforms to enable reliable inter-study comparisons.

## Discussion

This case underscores a fundamental technical issue in quantitative amyloid PET analysis. While the original CL standardization utilized the whole cerebellum as the reference [2], inclusion of cerebellar white matter voxels can lower cortical SUVRs and shift CL results downward. Several comparative studies have shown similar trends across amyloid tracers, with cerebellar gray matter yielding higher SUVRs and improved agreement with visual reads [8-10].

Such underestimation poses a critical risk in clinical practice. When CL values are interpreted in isolation, patients with visually evident amyloid deposition may be deemed ineligible for initiation of anti-amyloid therapy or, conversely, prematurely discontinued from treatment after apparent "normalization." This risk is amplified by national guidelines that specify numerical thresholds (e.g., <24.1 CL) to define negativity [4].

Clinicians should therefore prioritize expert visual assessment and treat quantitative results as complementary tools rather than absolute determinants. Transparent reporting of reference region definitions is essential for reproducibility and clinical reliability. Moreover, harmonization of processing pipelines across institutions is needed to prevent variability in SUVR and CL outcomes. Future studies may also incorporate plasma biomarkers, such as the amyloid-β 42/40 ratio and phosphorylated tau-217 (p-tau217), to adjudicate visually positive but quantitatively negative amyloid PET findings, thereby providing a more integrated framework for multimodal biomarker interpretation.

Finally, recent regulatory developments highlight the importance of interpretive consistency. In February 2025, Eli Lilly Nederland B.V. withdrew its application for the use of Amyvid^®^ to monitor treatment response, citing interpretive limitations in quantitative PET metrics [11]. This decision reflects ongoing uncertainty about the use of quantitative thresholds in clinical monitoring and reinforces the need for cautious application in real-world practice.

## Conclusions

This single case demonstrates how quantitative underestimation can occur in amyloid PET analysis when using the whole cerebellum as a reference region. Broader studies are warranted to determine the frequency and clinical impact of such discrepancies.

## References

[1] Clark CM, Schneider JA, Bedell BJ (2011). Use of florbetapir-PET for imaging beta-amyloid pathology. JAMA.

[2] Klunk WE, Koeppe RA, Price JC (2015). The Centiloid Project: standardizing quantitative amyloid plaque estimation by PET. Alzheimers Dement.

[3] Navitsky M, Joshi AD, Kennedy I (2018). Standardization of amyloid quantitation with florbetapir standardized uptake value ratios to the Centiloid scale. Alzheimers Dement.

[4] Fleisher AS, Chen K, Liu X (2011). Using positron emission tomography and florbetapir F18 to image cortical amyloid in patients with mild cognitive impairment or dementia due to Alzheimer disease. Arch Neurol.

[5] Matsuda H, Soma T, Okita K, Shigemoto Y, Sato N (2023). Development of software for measuring brain amyloid accumulation using (18) F-florbetapir PET and calculating global Centiloid scale and regional Z-score values. Brain Behav.

[6] (2025). Global Alzheimer’s Association Interactive Network (GAAIN). The Centiloid Project. https://www.gaain.org/centiloid-project.

[7] (2025). Standardizing Quantification of Amyloid PET Using the Centiloid Scale (MIMneuro White Paper). https://go.mimsoftware.com/literature/publications/access/centiliod-scale-white-paper.

[8] Brendel M, Högenauer M, Delker A, Sauerbeck J, Bartenstein P, Seibyl J, Rominger A (2015). Improved longitudinal [(18)F]-AV45 amyloid PET by white matter reference and VOI-based partial volume effect correction. Neuroimage.

[9] Chiao P, Bedell BJ, Avants B (2019). Impact of reference and target region selection on amyloid PET standard uptake value ratios in the Phase 1b PRIME study of aducanumab. J Nucl Med.

[10] Royse SK, Minhas DS, Lopresti BJ (2021). Validation of amyloid PET positivity thresholds in centiloids: a multisite PET study approach. Alzheimers Res Ther.

[11] (2025). Amyvid (florbetapir [18F])—withdrawal of the indication for monitoring treatment response in adults. https://www.ema.europa.eu/en/medicines/human/EPAR/amyvid.

